# A Phone Consultation Call Line to Support SBIRT in Pediatric Primary Care

**DOI:** 10.3389/fpsyt.2022.882486

**Published:** 2022-05-11

**Authors:** Sharon Levy, Alyssa Fuller, Shawn Kelly, Julie Lunstead, Elissa R. Weitzman, John H. Straus

**Affiliations:** ^1^Department of Pediatrics, Adolescent Substance Use and Addiction Program, Boston Children's Hospital, Boston, MA, United States; ^2^Department of Pediatrics, Harvard Medical School, Boston, MA, United States; ^3^Division of Adolescent and Young Adult Medicine, Boston Children's Hospital, Boston, MA, United States; ^4^Massachusetts Child Psychiatry Access Program, Beacon Health Options, Boston, MA, United States

**Keywords:** SBIRT, pediatric primary care, virtual care, substance use disorder (SUD), medication for addiction treatment, adolescent, substance use

## Abstract

**Background:**

Screening Brief Intervention Referral to Treatment (SBIRT) is recommended as a routine part of pediatric primary care, though managing patients with positive screens is challenging. To address this problem, the state of Massachusetts created a call line staffed by pediatric Addiction Medicine specialists to provide consultations to primary care providers and access to a behavioral health provider specially trained in managing adolescent substance use.

**Objective:**

To describe the uptake and outcomes of a consultation call line and virtual counseling for managing substance use disorders (SUD) in pediatric primary care.

**Methods:**

Service delivery data from consultations and counseling appointments were captured in an electronic database including substance, medication recommendations, level of care recommendations and number of counseling appointments completed for each patient. Summary data is presented here.

**Results:**

In all, there were 407 encounters to 108 unique families, including 128 consultations and 279 counseling visits in a one-year period. The most common substances mentioned by healthcare providers were cannabis (64%), nicotine (20%), alcohol (20%), vaping (9%) and opioids (5%). Management in primary care was recommended for 87 (68%) of the consultations. Medications for SUD treatment were recommended for 69 (54%) consultations including two for opioid use disorder.

**Conclusion:**

We found that both a statewide consultation call line and virtual counseling to support SBIRT in pediatric primary care were feasible. The majority of consultations resulted in recommendations for treatment in primary care.

## Introduction

Adolescents and young adults are the age group most likely to use psychoactive substances (1). Worldwide, more than 25% of adolescents use alcohol (2016 data) and more than 10% use nicotine (2008–2018 data) (2). Substance use (SU) during vulnerable windows of brain development that occur during adolescence and young adulthood is associated with adverse functional outcomes across domains (education/employment, family/social, health). As such, substance use (SU) is among the most important health risk behaviors for youth.

Healthcare professionals are called upon to help to mitigate the impact of substance use on youth, and screening for SU has long been recognized as important part of general healthcare. The World Health Organization (WHO) Alcohol, Smoking, and Substance Involvement Screening Test (ASSIST) has been validated in international settings, including with adolescents, and an accompanying manual has been developed to provide guidance on SU screening in primary care (3). In the United States (US), the American Academy of Pediatrics (AAP) recommends Screening, Brief Intervention, Referral to Treatment (SBIRT) for adolescents and has published detailed guidance on best practices (4). Pediatrician self-reported rates of SU screening and brief advice are high (5), though brief interventions and referrals are less common, and clinical expertise and community resources are significant barriers to SU treatment (6).

The US has a national shortage of pediatric mental health and behavioral care providers (7), limiting access to specialty care and increasing the pressure on primary care to expand services. Phone consultation programs that connect primary care providers with specialists are a promising approach to leverage the limited supply of child psychiatrists (8). These access lines can provide tele-consultation, training, resources, and referrals to providers (9). Nationwide, 45 such programs have been funded by the Health Resources and Services Administration (HRSA) (8). Children living in states with a consultation program have significantly greater mental health service use (7).

The Massachusetts Child Psychiatry Access Project (MCPAP) is the access line that serves the state of Massachusetts (10). More than 95% of pediatric primary care practices are enrolled in the MCPAP and the program provides mental health consultations to providers for more than 5,000 youth who receive care in a pediatric setting annually. There is no charge to any provider or patient for the consultation service. The majority of consultations come from pediatricians or pediatric nurse practitioners.

For this project, the MCPAP created a new phone consultation service staffed by pediatric Addiction Medicine specialists specifically to address questions regarding adolescent substance use from youth-facing primary care providers. This new service is available to all enrolled MCPAP practices (11). Here, we report usage metrics in order to assess the uptake and outcomes of this innovative statewide SBIRT support service.

## Methods

This report presents results of a retrospective audit of telephone consultations and virtual visits over a 1-year period, from Jan 1 through December 31, 2021.

### Program Description

The pediatric substance use consultation call line described here is available to any primary care provider within the state of Massachusetts with questions regarding adolescent substance use. Providers access the service by calling a central phone number that is shared with the MCPAP mental health consultation line. The service is available during normal business hours and is not designed to respond to emergencies. Trained administrators triage questions regarding substance use to the substance use line. New calls received by the substance use call intake coordinator are forwarded to the covering consultant.

At inception, providers were made aware of the new service through an email announcement, an article in the quarterly MCPAP newsletter sent to all registered users and a webinar open to all registered users. Providers who requested consults regarding substance use through the general MCPAP line were informed about ASAP-MCPAP by an intake coordinator and those consultations were forwarded to ASAP-MCPAP.

### Consultants

Consultants are all faculty members of the Adolescent Substance Use and Addiction Program at Boston Children's Hospital (ASAP) and Addiction Medicine Fellows. Primary specialties of the consultants include General Pediatrics, Developmental-Behavioral Pediatrics and Child and Adolescent Psychiatry. All consultants are board eligible, board certified or in training in Addiction Medicine or a nurse practitioner with extensive experience in adolescent substance use.

### Consultations

Consultants return all calls directly to the primary care provider that requests consultation. Most calls are returned on the same day. Addiction Medicine consultants did not speak directly with patients or families.

### Virtual Counseling

When appropriate, consultants recommend virtual counseling with the substance use BH provider. In general, patients were considered appropriate if they met the following criteria: 1) patient, parent or provider have concerns regarding substance use, 2) appropriate for outpatient therapy, 3) other forms of substance use counseling (integrated behavioral health or community referral) not available. Patients were considered ineligible for virtual care if referred to a higher level of care (i.e. outpatient substance use disorder treatment, intensive outpatient, residential treatment, etc.), if they were considered at high risk of withdrawal symptoms that require medical management (i.e. from alcohol or benzodiazepines) or if at high risk of overdose. In person assessment was recommended for patients with communication disorders for whom virtual care was not considered appropriate by the primary care provider, and patients for whom there were concerns of domestic violence.

A specially trained behavioral health (BH) provider conducted all appointments virtually using the Boston Children's Hospital (BCH) virtual visit platform. In this program, the behavioral health provider is a licensed independent clinical social worker. After each initial counseling appointment, the BH provider reviewed treatment recommendations with the referring PCP and entered an appointment encounter in the electronic database and a clinic note into the BCH electronic medical record. For this project, we analyzed data from every encounter completed between January 1 and December 31, 2021.

### Encounters

Each consultation request was entered into a secure electronic database that is compliant with the Health Insurance Portability and Accountability Act (HIPAA) of 1986. The encounter data fields include patient demographic information (age, sex, insurance plan, and de-identified member number), primary care practice, provider and encounter type, which were entered by an administrative assistant, and substance use concern, medication and outcome recommendations entered by the consultant (10). All identifiable patient information is encrypted and available only to consultants. The database is hosted by MCPAP, a third party contractor to the state of Massachusetts. Data summaries were provided by one of the authors (JS) who is the Founding Director of MCPAP. No personal health information was included in the database summary. This project was undertaken as a quality improvement effort and as such exempt from review by the Institutional Review Board.

#### Primary Concern

A list of 28 non-mutually exclusive concerns included eight substance use specific items (cannabis, nicotine, alcohol, vaping, opioids, stimulants, sedatives, non-specific substance).

#### Medications

Consultants selected from a list of 17 items including 14 commonly used psychopharmacologic agents, “Medication for Addiction Treatment (MAT)”, “other” and “no meds after encounter”. A free text field was available for “other” where specific medications were indicated.

#### Outcome

Consultants selected from nine outcomes describing recommended level of care, including: Primary Care Provider (PCP), bridge in primary care, therapist appointment – MCPAP (virtual ASAP therapist), therapist appointment non-MCPAP, ASAP, outpatient substance use program, Partial Hospital Program, Inpatient and Emergency Department. We considered recommendations for primary care provider, therapist appointment MCPAP, therapist appointment non-MCPAP as treated in primary care, while recommendations for outpatient substance use program, ASAP, partial hospital program and inpatient were considered specialty substance use treatment outside of primary care. We considered “bridge in primary care” to be a standalone category.

## Results

The ASAP-MCPAP program provided 407 encounters on behalf of 108 unique patients.

Patients represented in encounters were predominantly male (63%). The median age was 17 years (range 13–25 years).

Encounters were divided between consultation phone calls and virtual counseling visits as follows:

- 128 consultation calls from Addiction Medicine specialists to Primary Care Providers.
  ° 88 consultations were completed within a single call.
  ° 20 consultations were completed over two calls.
- 279 virtual counseling visits were provided to 36 patients (mean 7.8 visits, median 5 visits, IQR 2–11 visits per patient).

**Figure 1:**
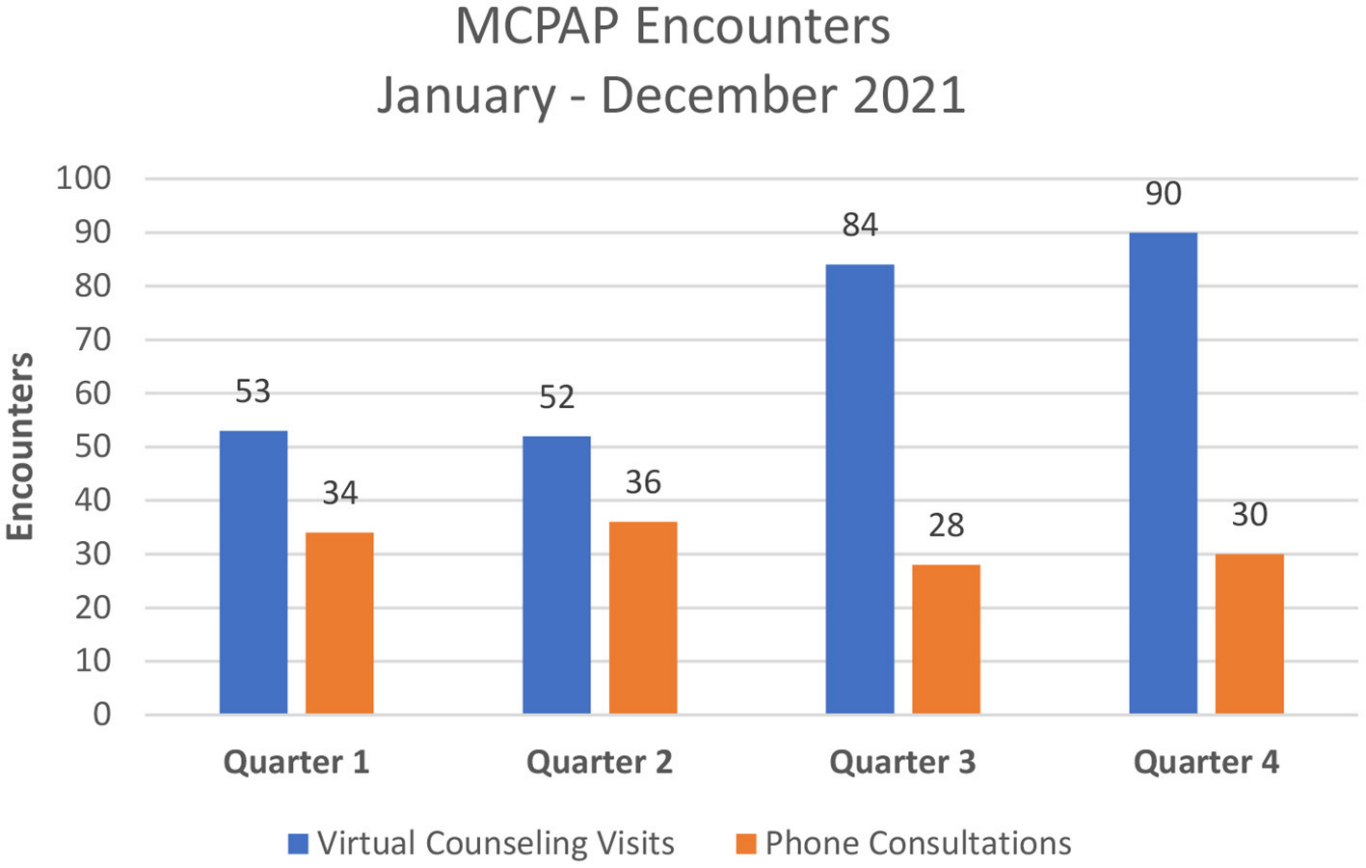
Number of virtual counseling visits and phone consultations per quarter.

Counseling was recommended as part of the consultation for 49 patients; 36 patients (73%) completed at least one counseling visit. Monthly counseling appointment volume steadily increased over the 1-year observation period (Figure 1).

The most common (non-mutually exclusive) substances mentioned by callers were cannabis (64%), nicotine (20%), alcohol (20%), vaping (9%) and opioids (5%). In 24 consultations (19%), callers did not identify the substance in question and for 9 consultations (7%) a mental health concern was considered primary and a substance was not listed. Recommendations for 87 consultations (68%) were for management in primary care, of those, 50 were also referred to an outpatient BH provider. Thirty-four consultations (27%) resulted in a recommendation for specialty substance use disorder treatment, including 27(21%), 2 (2%), and 5 (4%) to outpatient, partial hospital and inpatient, respectively. For five calls, the consultant recommended “bridge treatment in primary care” and the level of care recommendation was not recorded. Two calls were referred to an Emergency Department for further evaluation. Medications for SUD treatment were recommended for 69 patients (54%), including two for opioid use disorder (Table 1).

**Table 1:** Description of consultations.

	***N***	**%**
**Total Number of Consultations**	128	
Substance of Concern^*^	128	
Cannabis	82	64%
Nicotine	26	20%
Alcohol	25	20%
Vaping	12	9%
Opioid use	6	5%
Sedative use	4	3%
Stimulant use	4	3%
Non-specific substance	24	19%
Primary mental health concern/substance not listed	9	7%
**Outcomes**	128	
Treated in primary care	87	68%
Therapist appointment – non-MCPAP	1	1%
Therapist – MCPAP (virtual therapist)	49	38%
Bridge treatment with PCP	5	4%
Referred to specialty substance use treatment	34	27%
Outpatient 27 (21%)		
Partial program 2 (2%)		
Inpatient 5 (4%)		
Emergency department	2	2%
Medications^*^	128	
Medication for Addiction Treatment	2	2%
naloxone	2	2%
Naltrexone	8	6%
N-acetyl cysteine	52	41%
Nicotine Replacement Therapy	12	9%
Capsaicin	2	2%
No medication change	1	1%
No medication after encounter	59	46%

* *Not mutually exclusive*.

## Discussion

The unique pediatric substance use consultation and virtual counseling program described in this report provided 407 encounters on behalf of 108 unique families in a 1-year period. The volume of consultations decreased slightly between the first and fourth quarters of the observation period. We note that during the fourth quarter, Massachusetts experienced a surge in COVID-19 cases as the Omicron variant became prevalent. Additionally, during this period pediatric COVID vaccines became available. These two factors taxed pediatric healthcare resources and likely distracted attention from other issues including substance use. At the same time, virtual counseling appointments, which occurred outside of pediatric offices, increased over the course of the observation year. While there are no benchmarks to which to compare program volume, we consider our data an important demonstration of the utility of such a substance use consultation line.

Cannabis was the most common substance identified by callers as the reason for concern. This finding is consistent with reports that have found cannabis the most common cause for adolescents to seek substance use treatment both in Massachusetts (12) and other states (13–15). While national data has found that alcohol use is more common than cannabis use among adolescents, daily or near daily cannabis use is reported by 3.1% of teens (16). Some of the consultation calls were seeking treatment advice for cannabis hyperemesis syndrome or acute psychotic reactions, two acute medical problems related to cannabis use. These problems are increasing in frequency (17, 18) in association with policy changes that liberalize access to cannabis in Massachusetts and other states, and with increasing potency and variety of products available. These acute problems may cause patients to seek medical attention, and thereby shine a light on substance use in general for pediatric primary care providers.

We provided six consultations regarding opioid use, and recommended medications for opioid use disorder for two patients. Compared to adults, adolescents are far less likely to receive medication (19) for opioid use disorder (MAT), despite the effectiveness for youth (20, 21) and recommendations published by the AAP (22). Youth who do initiate treatment are more likely to be lost to follow up than older patients, (23). and it has been speculated that one of the reasons is that adult-centered substance use treatment programs do not meet youth's needs. Few OUD treatment programs for the general population provide services tailored for youth (24). Providing MAT in pediatric primary care can increase access to developmentally appropriate OUD treatment for youth, and is feasible (25). In this project, 73% of referred patients completed at least one counseling appointment and the mean number of counseling appointments was more than seven, which is similar to a recently published study that was conducted in a school-based setting (26). While the number of patients served by this project was small, providing MAT in pediatric primary care allows adolescents to access to treatment in the least restrictive setting. A consultation line can also be used to connect youth with OUD to other treatment settings where they can access MAT in combination with other treatments. Furthermore, consultation services may also help primary care providers appropriately address youth who report non-medical opioid use, but do not have an opioid use disorder. While rates of non-medical opioid use by high school students have decreased, approximately 2.3% of 12^th^ grade students currently report this behavior (16). These youth are at high risk of both acute (27) and long-term (28) consequences, thus attention to the behavior is warranted. Primary care, which offers adolescents an opportunity to have a confidential conversation with a trusted adult who can monitor their behavior over time, provides an excellent setting for this care.

In this project, the majority of recommendations were for treatment in primary care, with consultants offering advice on SU management. Delivering brief interventions in primary care is critical because even when specialists are available, many adolescents decline referral (29). Integrating behavioral health services within primary care allows adolescent patients easier access and better protects confidentiality as compared to appointments in an unfamiliar setting (30). Research on primary care SBIRT is promising: an evaluation in a large medical system found that SBIRT was associated with decreased substance use diagnoses and emergency department visits at 3-year follow up post-implementation (31, 32).

There is evidence that having specially trained BH providers do brief interventions may improve outcomes by decreasing rates of mental health disorders (33). More than half of the consultations recommending primary care management in this project were also referred for substance use counseling and all but one were referred to the program's own BH provider. In this model, counseling was delivered virtually, which may lower barriers for adolescent participation in substance use treatment (12). This program was designed to deliver coordinated care: healthcare professionals were the source of all referrals, received treatment recommendation summaries after each initial counseling visit and were encouraged to call the consultation line for support with medical components of care such as prescribing and drug testing as needed. The mean number of visits per patient was more than seven, representing substantial patient engagement, and supporting the acceptability of the program.

Standard brief interventions do not include the use of laboratory testing or medications to treat withdrawal or suppress cravings; adolescents are far less likely to receive effective treatment for substance use disorders (19, 34, 35) compared to adults. Consultants in this project recommended medication for substance use treatment, including nicotine replacement, naloxone, naltrexone and others, for more than half of all calls, suggesting that consultation service may be a good way to increase dissemination of medication for addiction treatment in youth.

Referral to treatment is the least studied aspect of adolescent SBIRT (30). Historically, few pediatric primary care providers refer adolescents with substance use concerns (36). While referrals and follow up appointments for problematic substance use may be becoming more common over time, healthcare providers report substantial barriers, (5). including unwillingness of adolescent patients to accept a referral or engage in care. Indeed, most adolescents with substance use disorders do not see their use as problematic (37). In this project, consultants recommended substance use specialty treatment in 27% of cases and provided support to PCP's including program information and suggestions for speaking with adolescent patients and their families about accepting a referral.

Our work has limitations. Consultants entered secondhand information reported by primary care providers and were unable to make diagnoses. However, information we recorded accurately represents the concerns presented by callers and as such, may be useful for planning efforts in other locales. We drew data from a clinical database and it is possible that different consultants used codes in the encounter form differently from one another, though we believe these differences are small as the group of consultants work together closely and communicated often. We do not know how many patients received the recommended medications or accepted referrals to substance use specialty treatment, nor do we have detailed patient-level outcome data to determine improvement. These are important quality measures that could be assessed in a future study. Finally, the scope of the project was small, and the work should be considered a pilot; data from a larger, scaled up version could be assessed in the future.

We conclude that in this project, provider to provider substance use consultation and provision of virtual substance use counseling enabled youth to access intervention for substance use within pediatric primary care. These services were offered through a statewide pediatric primary care access program. The infrastructure upon which these programs can be scaled exists because similar programs are available in most states and territories. Given the dearth of substance use treatment services for adolescents, innovative models such as this one may play an important role in building capacity.

## Data Availability Statement

The data analyzed in this study is subject to the following licenses/restrictions: The dataset used for this article reflects information about clinical encounters for current patients. Requests to access these datasets should be directed to sharon.levy@childrens.harvard.edu.

## Author Contributions

SL, AF, SK, JL, EW, and JS have participated in relevant study conception and design, acquisition of data, and analysis and data interpretation activities. All authors contributed to drafting or revising of the manuscript and have approved the manuscript as submitted.

## Funding

This project was supported in part by a subcontract through the American Academy of Pediatrics, on HRSA grant #1H7AMC37566-01-00.

## Conflict of Interest

The authors declare that the research was conducted in the absence of any commercial or financial relationships that could be construed as a potential conflict of interest.

## Publisher's Note

All claims expressed in this article are solely those of the authors and do not necessarily represent those of their affiliated organizations, or those of the publisher, the editors and the reviewers. Any product that may be evaluated in this article, or claim that may be made by its manufacturer, is not guaranteed or endorsed by the publisher.
